# Evaluating a transfer gradient assumption in a fomite-mediated microbial transmission model using an experimental and Bayesian approach

**DOI:** 10.1098/rsif.2020.0121

**Published:** 2020-06-24

**Authors:** Amanda M. Wilson, Marco-Felipe King, Martín López-García, Mark H. Weir, Jonathan D. Sexton, Robert A. Canales, Georgiana E. Kostov, Timothy R. Julian, Catherine J. Noakes, Kelly A. Reynolds

**Affiliations:** 1 Department of Community, Environment and Policy, Mel and Enid Zuckerman College of Public Health, University of Arizona, Tucson, AZ, USA; 2 School of Civil Engineering, University of Leeds, Leeds, UK; 3 School of Mathematics, University of Leeds, Leeds, UK; 4 Division of Environmental Health Sciences, College of Public Health, The Ohio State University, Columbus, OH, USA; 5 Department of Environmental Microbiology, Eawag, Swiss Federal Institute of Aquatic Science and Technology, CH-8600 Dübendorf, Switzerland; 6 Epidemiology and Public Health, Swiss Tropical and Public Health Institute, P.O. Box, CH-4002 Basel, Switzerland; 7 University of Basel, PO Box, CH-4003 Basel, Switzerland

**Keywords:** transfer efficiency, exposure, fomite, quantitative microbial risk assessment, phage, transmission

## Abstract

Current microbial exposure models assume that microbial exchange follows a concentration gradient during hand-to-surface contacts. Our objectives were to evaluate this assumption using transfer efficiency experiments and to evaluate a model's ability to explain concentration changes using approximate Bayesian computation (ABC) on these experimental data. Experiments were conducted with two phages (MS2, *Φ*X174) simultaneously to study bidirectional transfer. Concentrations on the fingertip and surface were quantified before and after fingertip-to-surface contacts. Prior distributions for surface and fingertip swabbing efficiencies and transfer efficiency were used to estimate concentrations on the fingertip and surface post contact. To inform posterior distributions, Euclidean distances were calculated for predicted detectable concentrations (log_10_ PFU cm^−2^) on the fingertip and surface post contact in comparison with experimental values. To demonstrate the usefulness of posterior distributions in calibrated model applications, posterior transfer efficiencies were used to estimate rotavirus infection risks for a fingertip-to-surface and subsequent fingertip-to-mouth contact. Experimental findings supported the transfer gradient assumption. Through ABC, the model explained concentration changes more consistently when concentrations on the fingertip and surface were similar. Future studies evaluating microbial transfer should consider accounting for differing fingertip-to-surface and surface-to-fingertip transfer efficiencies and extend this work for other microbial types.

## 1. Introduction

One mechanism by which viruses are spread and subsequently lead to exposure and disease is through human contacts with contaminated surfaces, or ‘fomites'. Contacts with fomites have been linked to microbial infection transmission [1–3], in part because of long survival times of pathogens in indoor environments [2,4,5]. The potential for transfer of viruses to and from hands during contacts with fomites has been demonstrated with viral tracer and viral transfer efficiency studies [6–9]. When contact between fomites and hands occurs, a portion of the microbial contaminant is transferred. This proportion is described quantitatively by transfer efficiency, or the fraction of a contaminant on an object that is transferred to another upon contact. This concept has been explored in chemical [10,11], microbial [7,9,12,13] and particle [14] contexts.

Transfer efficiency is influenced by environmental characteristics, the microorganisms and the transfer event. For example, low relative humidity (15–32%) generally results in lower transfer efficiencies than high relative humidity (40–65%) [9]. Transfer efficiencies have also been shown to be organism and surface dependent, where non-porous surfaces are associated with greater transfer efficiencies than porous surfaces [9]. They may also be dependent upon the direction of the transfer, e.g. surface-to-fingertip may be higher than fingertip-to-surface [7,15], and whether the hand in contact with the surface has been recently washed or not [7].

Transfer efficiencies are used to inform quantitative microbial risk assessments (QMRAs) and exposure models [16–18]. In equations used to estimate changes in microbial concentration on hands and on surfaces during hand-to-surface contacts, it has been assumed that there is a gradient of transfer where transfer efficiency is constant for both directions [16]. However, this has not been experimentally validated. While transfer efficiencies between hands and objects have typically not been identified as the most influential parameters in estimating risk [16,19], Greene *et al*. [20] demonstrated that not accounting for the effect of directionality on transfer efficiency in modelling can result in inaccuracies with sometimes more than 100% error [20]. The impact of transfer efficiency modelling assumptions on estimated exposures has not been assessed.

The primary study objective was to experimentally investigate a model assumption regarding a gradient of transfer between a fingertip and surface during fingertip-to-surface contact. The secondary objective was to use approximate Bayesian computation (ABC) to evaluate distributions for fingertip and surface swabbing efficiencies and transfer efficiency that best explain experimental findings and to demonstrate how these posterior distributions can be applied in QMRAs. This novel method has been applied in a recent transfer efficiency study to relate experimental data to microbial transfer models [21].

## 2. Methods

### 2.1. Transfer efficiency trials

Transfer efficiency trial methods were informed by Lopez *et al*. [9]. Three scenarios were investigated: one in which the surface (*s*) and the fingertip (*f*) had similar inoculation concentrations, theoretically resulting in similar viral concentrations before the fingertip-to-surface contact $\left(C_{k-1}^{f}≅C_{k-1}^{s}\right)$, one in which the fingertip inoculation concentration was greater than that of the surface $\left(C_{k-1}^{f}>C_{k-1}^{s}\right)$ and one in which the fingertip concentration was less than that of the surface $(C_{k-1}^{f}<C_{k-1}^{s});\;k-1$ denotes the before-contact state. Two phages, MS2 (ATCC 15597-B1) and *Φ*X174 (ATCC 13706-B1), were used to track viral transfer from the surface to the fingertip and from the fingertip to the surface within the same trial. For each scenario, three trials were conducted in which MS2 was used to inoculate the fingertip and *Φ*X174 was used to inoculate the surface for the same trial. Three other trials were conducted for each concentration scenario in which *Φ*X174 was used to inoculate the fingertip and MS2 was used to inoculate the surface. A porous (unsealed), hard ceramic tile was used for the fingertip-to-surface contacts. Before trials, ceramic tiles were wrapped in tinfoil and autoclaved. Viruses in tryptic soy broth were filtered with 0.2 μm filters (Syringe Filter; VWR^®^, Radnor, PA) on the day of the trial to remove any large viral aggregates that could create discrepancies between total viruses quantified in before-contact and after-contact samples.

Negative controls were conducted before each trial, where a fingertip and an autoclaved ceramic tile not to be inoculated were each swabbed with 1 ml letheen broth swabs (Swab-Sampler with 1 ml letheen broth; 3M™, Maplewood, MN). The swabs were processed for MS2 and *Φ*X174 to ensure that the only MS2 and *Φ*X174 particles detected were a result of the trial and not due to their presence in the experimental environment or contamination from previous trials. Trials for which any of the negative controls were positive were excluded or re-conducted. Temperature and relative humidity for each trial were measured using a digital hygrometer (Traceable^®^; VWR) and recorded for later comparison of environmental conditions between trials in which MS2 was used on the fingertip and *Φ*X174 on the surface or vice versa. Trials were conducted with the same right-handed participant to limit variability related to finger size. The participant was instructed to contact the tile for 1 s and practised achieving a contact force between 700 and 1500 g on the scale; the maximum point value observed during the contact was reported.

Both index fingertips were inoculated with 10 µl of the virus. The pipette tip for inoculating the fingertip was used to spread the inoculation liquid evenly over the surface of the fingertip up to the first knuckle from the fingertip. Two separate 10 µl dots of virus were placed on the autoclaved ceramic tile. The pipette tip was used to scrape the inoculation liquid into an approximate elliptical fingertip shape in order to prevent excessive waiting for drying. The fingertips and the tile were inoculated consecutively. Initial concentrations on the tile ranged from 2.73 × 10^0^ to 7.70 × 10^7^ plaque-forming units (PFU) cm^−2^, while initial concentrations on the fingertip ranged from 1.00 × 10^2^ to 1.10 × 10^8^ PFU cm^−2^. These initial concentration ranges were large because inoculations were diluted specifically to achieve a variety of ratios in before-contact log_10_ concentrations on the fingertip for transfer to the tile surface.

When all inoculations were visibly dry, the trial was initiated. The left fingertip was swabbed to represent the concentration on the fingertip before the fingertip-to-surface contact and after inoculum drying. The fingertip pad was swabbed up to the first knuckle, consistent with the inoculation area, with a back and forth motion, rolling the swab. One inoculation dot on the tile was swabbed to represent the concentration on the tile surface before the fingertip-to-surface contact and after inoculum drying. The inoculation area was swabbed in a back and forth, circular motion. The participant then made a 1 s contact using the right fingertip with the un-swabbed inoculated area on the ceramic tile. Following the contact, the right fingertip and the inoculation area on the ceramic tile that was contacted were swabbed to measure the viral concentrations after the fingertip-to-surface contact with swabbing motions and areas described above. All swabs were processed for both viruses using dilution series and the double agar overlay method [22], assayed in duplicate. If the virus not used for inoculation of the fingertip or surface of that particular trial was detected in before-contact samples, the trial was re-conducted. The hosts used to process for MS2 and *Φ*X174 were *Escherichia coli* strain C-3000 (ATCC 15597) and *E. coli* strain C (ATCC 13706), respectively. All samples were incubated for 24 h at 37°C, and plaques were enumerated. When possible, the plates with a dilution yielding plaque counts between 20 and 200 were used to represent the viral concentration for that sample and counts for the two duplicates of that dilution were averaged. If one of the duplicate plates had no plaques, an average of 0 and the count on the other plate was taken. No substitution or multiple imputation methods were used to replace these censored values because the trials did not all belong to the same distribution of ‘before' (*k* − 1) or ‘after' (*k*) fingertip-to-contact concentrations.

Following trials, hands were washed with warm water and non-antimicrobial soap. The same fingertip was used for all trials. A single trial was conducted per day to avoid cross contamination. The surface area of contact was measured by putting non-toxic ink on the fingertip and pressing for 1 s with this fingertip on paper. This was scanned, and the surface area in pixels was converted to centimetres squared using an open source photo and graphics editor software, GNU Image Manipulation Program (GIMP; The GIMP Development Team).

### 2.2. Recoverable transfer efficiency calculations

Recoverable transfer efficiencies [13] for total virus (MS2 and *Φ*X174 combined) were calculated for transfer to the fingertip and to the surface, using equations based on recoverable transfer efficiency calculations from Pitol *et al*. [13] using equations (2.1) and (2.2),

2.1 $$\begin{array}{rl} & TE_{\mathrm{recoverable}}^{f}\\ & \;=\frac{C_{\mathrm{observed},k,\mathrm{MS}2}^{f}+C_{\mathrm{observed},k,\Phi X174}^{f}}{C_{\mathrm{observed},k,\mathrm{MS}2}^{f}+C_{\mathrm{observed},k,\Phi X174}^{f}+C_{\mathrm{observed},k,\mathrm{MS}2}^{s}+C_{\mathrm{observed},k,\Phi X174}^{s}} \end{array}$$

and

2.2 $$\begin{array}{rl} & TE_{\mathrm{recoverable}}^{s}\\ & \;=\frac{C_{\mathrm{observed},k,\mathrm{MS}2}^{s}+C_{\mathrm{observed},k,\Phi X174}^{s}}{C_{\mathrm{observed},k,\mathrm{MS}2}^{f}+C_{\mathrm{observed},k,\Phi X174}^{f}+C_{\mathrm{observed},k,\mathrm{MS}2}^{s}+C_{\mathrm{observed},k,\Phi X174}^{s}}, \end{array}$$

where $TE_{\mathrm{recoverable}}^{f}$ is the recoverable fingertip transfer efficiency (fraction), $TE_{\mathrm{recoverable}}^{s}$ is the recoverable surface transfer efficiency (fraction) and $C_{\mathrm{observed},k,\mathrm{MS}2}^{f}\;\mathrm{and}\;C_{\mathrm{observed},k,\Phi X174}^{f}$ are the observed MS2 and *Φ*X174 concentrations on the fingertip, respectively, after the fingertip-to-surface contact. $C_{\mathrm{observed},k,\mathrm{MS}2}^{s}$ and $C_{\mathrm{observed},k,\Phi X174}^{s}$ are the observed MS2 and *Φ*X174 concentrations on the surface, respectively, after the fingertip-to-surface contact.

These can also be calculated in terms of PFU cm^−2^. It was assumed that sampled surface areas corresponded to the contact surface area. Therefore, dividing each concentration by the fingertip surface area and then dividing the sum of concentrations to calculate recoverable transfer efficiency would cancel the initial division by the contact surface area. This was therefore simplified by keeping sample concentrations in PFU ml^−1^ for transfer efficiency calculations.

In this case, the sum of final concentrations in the denominator represents the total amount of virus that could have been transferred, and before-contact concentrations were not used in the denominator because of potential differences in total virus concentrations in before and after samples [13]. The numerator expresses the total amount of virus present on the object of interest after the contact [13]. The division of the numerator by the denominator, therefore, represents the fraction of total virus present on the object of interest after the fingertip-to-fomite contact [13].

### 2.3. Investigating the gradient of transfer assumption with a logistic curve

A sigmoid curve has been used to describe the relationship between the logarithm of a concentration and a particular response, sometimes referred to as ‘mass action' [23]. To evaluate whether a transfer gradient was observed experimentally, a logistic curve, a type of sigmoid curve, was fitted to the recoverable transfer efficiency of the fingertip ($TE_{\mathrm{recoverable}}^{f}$) as a function of the log_10_-transformed before-contact concentration on the fingertip divided by the before-contact concentration on the surface ($C_{k-1}^{f}/C_{k-1}^{s}$). This was done using the R [24] package *sicegar* [25] to evaluate the hypothesis that transfer efficiencies, in this case recoverable transfer efficiencies, were a function of ratios of before-contact concentrations between two surfaces in contact (equation (2.3)),

2.3 $$\;TE_{\mathrm{recoverable}}^{f}(x)=\frac{TE_{\mathrm{recoverable},\max}^{f}}{1+{\text{e}}^{-a_{1}(x-\mathrm{lo}g_{10}{\left(C_{k-1}^{f}/C_{k-1}^{s}\right)}_{\mathrm{mid}})}}.$$

Here, $TE_{\mathrm{recoverable},\max}^{f}$ is the maximum recoverable fingertip transfer efficiency, $\text{lo}{\text{g}}_{10}{\left(C_{k-1}^{f}/C_{k-1}^{s}\right)}_{\mathrm{mid}}$ is the mid-log_10_-transformed ratio where the inflection occurs, *x* represents the$\;\mathrm{lo}{\text{g}}_{10}(C_{k-1}^{f}/C_{k-1}^{s})$ and *a*_1_ is the shape parameter referred to as ‘growth rate'. Owing to limitations with the package's ability to fit to negative ratio values, log_10_-transformed ratios were scaled upwards by adding the minimum magnitude to all values. During interpretation of the curves, the mid-value $\left(\text{lo}{\text{g}}_{10}{\left(C_{k-1}^{f}/C_{k-1}^{s}\right)}_{\mathrm{mid}}\right)$ was adjusted to reflect this.

### 2.4. Mathematical model and parameter estimation

Equations used by Julian *et al*. [16] to describe changes in microbial concentration on the fingertip and surface following a fingertip-to-surface contact were used to predict changes in viral concentrations observed in experimental trials. This model is a deterministic recurrence relation or linear difference equation that describes a gradient of microbial transfer between two objects in contact and has been used in the context of rotavirus exposure assessment, consistent with bacteriophage used as enteric virus surrogates in this study [16]. In the original Julian *et al*. [16] model, parameters include viral concentration on the surface and hand, inactivation constants of the virus on the hand or on the surface, transfer efficiency, the fraction of the hand in contact with the surface, the surface area of the surface in contact and the surface area of the hand.

Since changes in viral concentration on the fingertip and the surface area of contact on the surface were estimated in this study, hand surface area, surface area of the surface and fraction of the hand in contact were not relevant parameters. Equations were adjusted where viral die-off was removed owing to negligible expected loss of MS2 and *Φ*X174 during the experimental trials, where die-off rates for MS2 may be as low as 5.1 × 10^−5^ to 1.0 × 10^−4^ min^−1^ [26,27]. Therefore, variables from the original Julian *et al*. [16] model that were included in this adapted model were transfer efficiency and viral concentrations on the fingertip and surface. Before-contact concentrations of virus on the surface and on the fingertip from the experiment (*n* = 16) were used in this analysis, and swabbing efficiencies were accounted for so that the predicted concentration represented the true virus concentrations on the fingertip and surface, not just the detectable portion. The fraction of contact surface area was removed from the original equation because of the assumption that the contact area and the contaminated surface area are approximately equal. Detectable virus concentrations on the fingertip and surface were calculated by the following equations (equation (2.4)–(2.5)):

2.4 $$C_{\mathrm{predicted},k}^{f}=\frac{C_{\mathrm{observed},k-1}^{f}}{S^{f}}-\lambda \times \left(\frac{C_{\mathrm{observed},k-1}^{f}}{S^{f}}-\frac{C_{\mathrm{observed},k-1}^{s}}{S^{s}}\right)$$

and

2.5 $$C_{\mathrm{predicted},k}^{s}=\frac{C_{\mathrm{observed},k-1}^{s}}{S^{s}}-\lambda \times \left(\frac{C_{\mathrm{observed},k-1}^{s}}{S^{s}}-\frac{C_{\mathrm{observed},k-1}^{f}}{S^{f}}\right).$$

In equations (2.4) and (2.5), $C_{\mathrm{predicted},k}^{f}$ is the predicted PFU/fingertip (*f*) after the fingertip-to-surface contact, $C_{\mathrm{predicted},k}^{s}$ is the predicted PFU/surface (*s*) at the inoculation area after the fingertip-to-surface contact, $C_{\mathrm{observed},k-1}^{f}$ is the experimentally observed PFU/fingertip (*f*) before the fingertip-to-surface contact and $C_{\mathrm{observed},k-1}^{s}$ is the experimentally observed PFU/surface (*s*) at the inoculation area before the fingertip-to-surface contact. While log_10_ concentrations are used to compare model-predicted and experimentally observed concentrations, these equations (equations (2.4) and (2.5)) do not use log_10_-transformed concentrations. *S^f^* is the swabbing efficiency (fraction) for the sampling of the fingertip, *S^s^* is swabbing efficiency (fraction) for the sampling of the surface and *λ* is the transfer efficiency.

Transfer efficiency (*λ*) in the gradient transfer model is relatable to the recoverable transfer efficiency $\left(TE_{\mathrm{recoverable}}^{f}\;\text{or}\;TE_{\mathrm{recoverable}}^{s}\right)$ discussed in §2.2 when only one of the two objects in contact (either the fingertip or surface) is contaminated. In this case, transfer efficiency (*λ*) is equal to the fraction of total virus available for transfer that is expected to be on the fingertip or surface after the contact. However, when both surfaces are contaminated, transfer efficiency (*λ*) describes the fraction of the difference in viral concentration between the fingertip and fomite to be transferred (equations (2.4) and (2.5)). In this study, all cases involve contact between contaminated fingertips and contaminated surfaces.

Prior distributions were included for fingertip swabbing efficiency, surface swabbing efficiency and transfer efficiency, where, for each iteration, single values from distributions for fingertip swabbing efficiency, surface swabbing efficiency, transfer efficiency and before-contact viral concentrations on the fingertip and surface were randomly sampled. Ten million iterations were run, and the combinations of parameters with the 0.1% lowest Euclidean distances were used to create posterior distributions of these parameters, each with 10 000 values. A large number of iterations are typically needed with the ABC approach in order to be more restrictive in defining posterior distributions, yielding an informative number, in this case 10 000, of best combinations of model inputs, where there is a large number of possible combinations of values from the prior distributions [28]. The Euclidean distance equation was used to measure the distance between the model-estimated and the experimentally measured concentrations on the fingertip and surface after the contact. The sum of the square errors, as is used here (equation (2.6)), has been used in other ABC research [21,28]. Model-estimated concentrations on the fingertip represent the true number of virus particles on the fingertip, while those experimentally measured represent the number of detected virus particles influenced by swabbing efficiency. Experimental concentrations were therefore adjusted by multiplying by the randomly sampled fingertip and surface swabbing efficiencies, respectively, from their prior distributions to estimate detected viral concentrations on the fingertip (equation (2.6)),

2.6 $$\begin{array}{rl} & \delta (C_{\mathrm{predicted},k}^{f},C_{\mathrm{observed},k}^{f},C_{\mathrm{predicted},k}^{s},C_{\mathrm{observed},k}^{s})=\\ & \sqrt{\sum_{\;j=1}^{16}{\left(\text{lo}{\text{g}}_{10}(C_{\mathrm{predicted},k,j}^{f}\cdot S^{f})-\text{lo}{\text{g}}_{10}(C_{\mathrm{observed},k,j}^{f})\right)}^{2}+{\left(\text{lo}{\text{g}}_{10}(C_{\mathrm{predicted},k,j}^{s}\cdot S^{s})-\text{lo}{\text{g}}_{10}(C_{\mathrm{observed},k,j}^{s})\right)}^{2}}. \end{array}$$

In equation (2.6), *j* represents the trial number, of which there is a total of 16.

To evaluate whether the model's accuracy or consistency in predictions was affected by ratios of before-contact concentrations, differences between after-contact predicted and experimental log_10_ concentrations on the fingertip were plotted against ratios of the before-contact fingertip and surface concentrations. A Bland–Altman plot was used to evaluate the model's agreement with experimental data by plotting differences between the median of all after-contact concentrations in the posterior distribution estimated for each of the 16 experimental trials and the mean of the experimental concentration and the median of the predicted concentrations for that experimental datum point.

### 2.5. Quantitative microbial risk assessment application

To demonstrate how the ABC method can be used in future studies to investigate fomite-mediated transmission in infection risk estimates, posterior distributions were used in a Monte Carlo simulation of 10 000 iterations to estimate viral concentration on a fingertip after a single fingertip-to-surface contact for scenarios where the concentration on the fingertip and on the surface ranges from 10^−2^ to 10^2^ PFU/surface or /fingertip,

2.7 $$C_{\mathrm{predicted},k}^{f}=C_{k-1}^{f}-\lambda (C_{k-1}^{f}-C_{k-1}^{s}).$$

In equation (2.7), $C_{\mathrm{predicted},k}^{f}$ is the predicted PFU/fingertip (*f*) after the fingertip-to-surface contact, $C_{k-1}^{f}$ is the concentration on the fingertip (*f*) before the fingertip-to-surface contact, $C_{k-1}^{s}$ is the concentration on the surface (*s*) of the area of contact before the fingertip-to-surface contact and *λ* is the transfer efficiency (fraction) from the posterior distribution. A single fingertip-to-surface contact was evaluated as opposed to multiple contacts for consistency with the experimental approach of this study in which a single fingertip-to-surface was used. Single fingertip-to-surface contact exposure scenarios have been used to inform potential risk targets and cleanliness goals for surfaces in healthcare environments [29,30]. Here, it is assumed that the fingertip surface area is equal to the surface area of contact for consistency with the equations evaluated using the ABC method.

While the posterior transfer efficiencies (*λ*) were randomly sampled without replacement in the QMRA application of this study, a distribution was fitted to the posterior distribution for future research applications. Candidate distributions included Lognormal, Gamma, Beta and Weibull, as these distributions have been previously included as candidate distributions for describing transfer efficiencies [31]. Kolmogorov–Smirnov and *χ*^2^ test statistics, the Akaike information criterion (AIC) and visual evaluation of distribution fits were used to compare candidate distribution fits, where larger test statistics and smaller AIC values were favourable [31].

Swabbing efficiencies were not incorporated here because, in estimating exposures and subsequent infection risks, it was assumed that before-contact concentrations on the surfaces of the fingertips were not detectable virus but rather ‘true' or present concentrations of virus. Dose was computed so that the probability of infection for a single fingertip-to-fomite contact followed by a single fingertip-to-mouth contact can be calculated, where dose is the number of ingested viral particles from the finger-to-mouth contact, $C_{\mathrm{predicted},k}^{f}$ is the viral concentration on the fingertip following the fingertip-to-mouth contact and *TE*_mouth_ is the hand-to-mouth transfer efficiency (equation (2.8)),

2.8 $$\text{Dose}=C_{\mathrm{predicted},k}^{f}\cdot TE_{\mathrm{mouth}}.$$

The surface area of contact is not accounted for, as it is assumed that the fingertip area used in the fingertip-to-surface contact is used for the fingertip-to-mouth contact (equation (2.8)). Sources for distributions of parameters used in estimating exposure can be seen in table 1.

**Table 1.** Parameters, their distributions and sources.

parameter	variable	distribution	source
*prior distributions of ABC analysis*			
transfer efficiency	*λ*	uniform (0.0001, 0.406)	Lopez *et al.* [9]
surface swabbing efficiency	*S*^s^	uniform (0, 1)	assumed
fingertip swabbing efficiency	*S^f^*	uniform (0, 1)	assumed
*parameters for QMRA application*			
transfer efficiency	*λ*	lognormal^c^ (mean log = −5.07, s.d. log = 0.11)	this study
transfer efficiency (hand-to-mouth)	*TE*_mouth_	normal^a^ (*μ* = 0.3390, *σ* = 0.1)	Rusin *et al.* [12]; Julian *et al.* [16]
concentration on fingertip before hand-to-fomite contact	$C_{k-1}^{f}$	uniform (PFU/fingertip) (min = 10^−2^, max = 10^2^)	assumed
concentration on surface before hand-to-fomite contact	$C_{k-1}^{s}$	uniform (PFU/area of contacted surface) (min = 10^−2^, max = 10^2^)	assumed
dose–response curve parameters^b^	*α*	23.4 ± 366.9 median = 0.26 (min = 0.09, max = 9452.7)	this study
	*N*_50_	8.08 ± 6.64 median = 6.30 (min = 1.02, max = 1.3 × 10^2^)	this study

^a^Distribution left truncated at 0 and right truncated at 1.

^b^The α and *N*_50_ values are to be used in pairs. Paired values are available in the electronic supplementary material along with code for generating α and *N*_50_ pairs. The mean ± s.d., median, minimum and maximum are provided here.

^c^In this study, the posterior distribution was randomly sampled directly. However, this distribution fit (right truncated at 1) could be used as an alternative.

Infection risk was then estimated by assuming a fingertip-to-mouth contact directly following the fingertip-to-surface contact. Infection risk was modelled for rotavirus because it is an enteric virus, as are the viruses in the experimental study. Rotavirus has been used in a previous QMRA as a conservative risk estimate because of its relatively low infectious dose (6.17 viral particles) [32,33]. The dose–response relationship was modelled as an approximate beta-Poisson, where *P*_infection_(*d*) is the probability of infection for a given dose (number of viral particles), *d* (equation (2.9)). In equation (2.9), *α* and *N*_50_ are dose–response curve parameters [34],

2.9 $$P_{\mathrm{infection}}(d)=1-{\left[1+d\frac{\left(2^{1/\alpha }-1\right)}{N_{50}}\right]}^{-\alpha }.$$

Infection risks were estimated with bootstrapped combinations of *α* and *N*_50_ values retrieved from an approximate beta-Poisson dose–response curve fitted to a rotavirus human infectious dose study using a maximum-likelihood estimation approach [35] (electronic supplementary material, figure S1).

### 2.6. Sensitivity analysis

Estimated infection risks were plotted against respective input parameters and infection risk. Spearman correlation coefficients were calculated, and a global variance-based sensitivity analysis was conducted using Sobol' indices [36]. First-order and total Sobol' indices were estimated using the sobol2007 function from the R package, *sensitivity* [37], where the first-order indices represent the individual contribution of each input to the variance in the output, which in this case is infection risk, and the total effect indices represent the contribution of the input taking into account its interactions with other input variables to the variance in the output.

## 3. Results

### 3.1. Experimental findings

The number of viral particles detected in processing 1 ml of letheen broth in each swab represented the number of particles per swabbed contact area. The surface area of the fingertip used in all trials was 1.83 cm^2^. Two of the 18 trials were discarded owing to errors in trial recording and a detection issue. Initial concentrations on the porous tile surface ranged from 5.00 × 10^0^ to 1.41 × 10^8^ PFU per inoculation area, while initial concentrations on the fingertip ranged from 1.83 × 10^2^ to 2.02 × 10^8^ PFU cm^−2^ (table 2). The ratios of before-contact (*k* − 1) concentrations on the fingertip (*f*) and surface (*s*) $\left(C_{\mathrm{observed},k-1\;}^{f}/C_{\mathrm{observed},k-1}^{s}\right)$ ranged from 4.80 × 10^−5^ to 9.60 × 10^4^ (table 2).

**Table 2.** Geometric mean (geomean) ± geometric standard deviations (geos.d.) and ranges (min, max) of concentrations (PFU/contact surface area) and recoverable transfer efficiencies.

		fingertip > surface (*n* = 8)	fingertip < surface (*n* = 8)	all trials (*n* = 16)
before-contact concentration of fingertip	geomean ± geos.d.	3.14 × 10^6^ ± 1.21 × 10^1^	3.31 × 10^4^ ± 2.45 × 10^1^	3.22 × 10^5^ ± 3.78 × 10^1^
	min, max	3.35 × 10^5^, 2.02 × 10^8^	1.83 × 10^2^, 7.70 × 10^5^	1.83 × 10^2^, 2.02 × 10^8^
before-contact concentration of surface	geomean ± geos.d.	2.32 × 10^4^ ± 1.26 × 10^2^	7.45 × 10^6^ ± 4.75 × 10^0^	4.16 × 10^5^ ± 9.72 × 10^1^
	min, max	5.00 × 10^0^, 3.35 × 10^6^	2.30 × 10^6^, 1.41 × 10^8^	5.00 × 10^0^, 1.41 × 10^8^
after-contact concentration of fingertip	geomean ± geos.d.	2.98 × 10^6^ ± 1.05 × 10^1^	1.45 × 10^5^ ± 5.43 × 10^0^	6.58 × 10^5^ ± 1.25 × 10^1^
	min, max	4.45 × 10^5^, 2.07 × 10^8^	1.12 × 10^4^, 1.67 × 10^6^	1.12 × 10^4^, 2.07 × 10^8^
after-contact concentration of surface	geomean ± geos.d.	7.69 × 10^4^ ± 2.40 × 10^1^	5.62 × 10^6^ ± 6.29 × 10^0^	6.58 × 10^5^ ± 2.84 × 10^1^
	min, max	1.15 × 10^3^, 2.54 × 10^6^	9.16 × 10^5^, 1.71 × 10^8^	1.15 × 10^3^, 1.71 × 10^8^
ratio of before-contact fingertip-to-surface concentrations	geomean ± geos.d.	1.35 × 10^2^ ± 2.71 × 10^1^	4.45 × 10^−3^ ± 1.51 × 10^1^	7.76 × 10^−1^ ± 4.36 × 10^2^
	min, max	3.44 × 10^0^, 9.60 × 10^4^	4.8 × 10^−5^, 6.69 × 10^−2^	4.8 × 10^−5^, 9.60 × 10^4^
recoverable fingertip transfer efficiency	geomean ± geos.d.	9.35 × 10^−1^ ± 1.10 × 10^0^	2.46 × 10^−2^ ± 2.97 × 10^0^	1.51 × 10^−1^ ± 7.55 × 10^0^
	min, max	7.59 × 10^−1^, 9.98 × 10^−1^	7.61 × 10^−3^, 2.07 × 10^−1^	7.60 × 10^−3^, 9.98 × 10^−1^

While it was intended that ratios of before-contact concentrations for fingertip and surface would span a range of magnitudes where, in some cases, the before-contact concentrations on the fingertip and surface would be of the same order of magnitude, differences between inoculum concentrations and before-contact concentrations were observed. This may be explained by differences in swabbing efficiencies for skin and the ceramic tile surface. The before-contact fingertip concentration was greater than that of the surface for eight out of 16 trials, with ratios of fingertip-to-surface concentration $\left(C_{k-1}^{f}/C_{k-1}^{s}\right)$ ranging from 3.44 × 10^0^ to 9.60 × 10^4^. For the other eight out of 16 trials, the before-contact concentration on the surface was greater than that of the fingertip, with ratios of fingertip-to-surface concentration ranging from 4.80 × 10^−5^ to 6.67 × 10^−2^. For all trials, final concentrations on the fingertip $\left(C_{k}^{f}\right)$ ranged from 1.12 × 10^4^ to 2.07 × 10^8^ PFU/fingertip, and final concentrations on the surface $\left(C_{k}^{s}\right)$ ranged from 1.15 × 10^3^ to 1.71 × 10^8^ PFU/inoculation area. Recoverable fingertip transfer efficiency$\;(TE_{\mathrm{recoverable}}^{f})\;$ranged from 7.60 × 10^−3^ to 9.98 × 10^−1^. For trials in which the before-contact concentration on the fingertip was greater than that of the surface, this ranged from 7.59 × 10^−1^ to 9.98 × 10^−1^; for trials in which the before-contact concentration on the fingertip was smaller, this ranged from 7.61 × 10^−3^ to 2.07 × 10^−1^. Relative humidity (%) and temperature (°C) ranged from 13% to 31% and from 17.4°C to 23.6°C during the experimental work with no notable differences between trials using MS2 on the fingertip and those with *Φ*X174 on the fingertip. Experimental results are summarized in table 2.

### 3.2. Logistic curve fit

**Figure 1.**
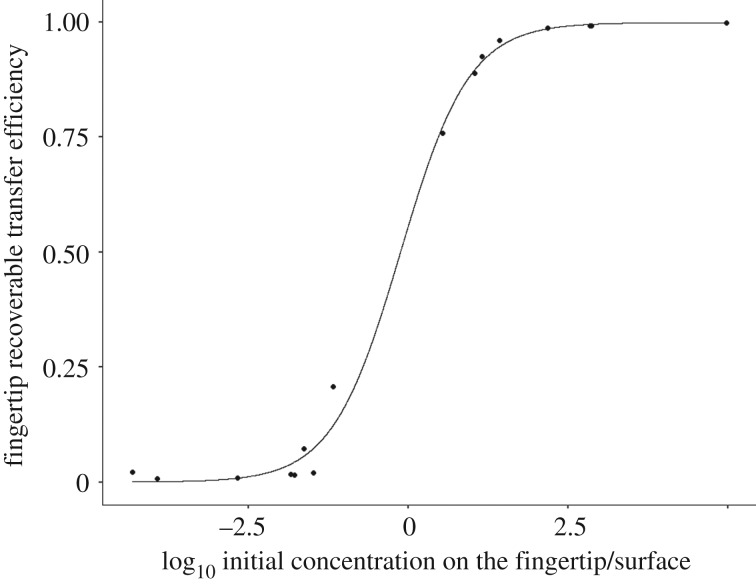
Recoverable transfer efficiency $(C_{\mathrm{observed},k}^{f}/C_{\mathrm{observed},k}^{f}+C_{\mathrm{observed},k}^{s})\;$ for the fingertip versus log_10_ ratio of contamination between the fingertip and surface $\left(\text{lo}{\text{g}}_{10}(C_{\mathrm{observed},k-1}^{f}/C_{\mathrm{observed},k-1}^{s})\right)$ with a logistic fit.

The fit curve can be described by equation (3.1), depicted in figure 1, where *x* = log_10_-transformed $C_{k-1}^{f}/C_{k-1}^{s}$,

3.1 $$TE_{r\mathrm{ecoverable}}^{f}=\frac{0.99}{1+{\text{e}}^{-1.86(x-0.12)}}.$$

The numerator, 0.99, represents the maximum value approached. We would expect this value to be 1. However, the maximum value observed experimentally was 0.99. The midpoint where the expected shift occurs is −0.12, which relates to a ratio in concentration of 10^−0.12^. This is relatively close to 10^0^, the point at which the initial concentrations on both objects are equal. A midpoint with a negative value suggests preferential adsorption of virus to the finger relative to the surface. The value 1.86 relates to what is usually referred to as the logistic ‘growth rate.' Within this context, this represents the logistic rate of increase in recoverable transfer efficiency relative to the ratio of the initial concentrations. When the concentration of viruses on the fingertip is much larger than that on the surface, the recoverable transfer to the fingertip is large. As the concentrations on the fingertip and the surface become closer, the recoverable transfer to the fingertip decreases more steeply (figure 1).

### 3.3. Approximate Bayesian computation

**Figure 2.**
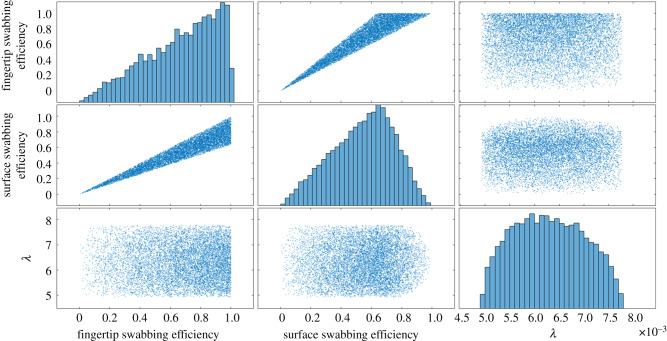
Posterior distributions containing values of transfer efficiency (*λ*), surface swabbing efficiency and fingertip swabbing efficiency that resulted in the 0.1% smallest Euclidean distances for 1 × 10^7^ iterations.

Comparisons of prior and posterior distributions offered insights into swabbing efficiency (*S^f^* and *S^s^*) and transfer efficiency (*λ*) values that best explained experimentally measured viral concentrations on the fingertip and surface after the fingertip-to-surface contact ($C_{k}^{f}$ and $C_{k}^{s}$), as there were differences in posterior distribution shape and central tendencies for fingertip and surface swabbing efficiency despite sharing a prior distribution (figure 2). The posterior distribution for fingertip swabbing efficiency (*S^f^*) ranged from 8.7 × 10^−3^ to 1.0 × 10^0^ with a median and standard deviation (s.d.) of 7.1 × 10^−1^ and 2.4 × 10^−1^, respectively. The posterior distribution for surface swabbing efficiency (*S^s^*) ranged from 7.8 × 10^−3^ to 9.9 × 10^−1^ with a median of 5.6 × 10^−1^ and a s.d. of 2.0 × 10^−1^, respectively. The posterior distribution for transfer efficiency (*λ*) ranged from 4.9 × 10^−3^ to 7.8 × 10^−3^ with a median of 6.3 × 10^−3^ and a s.d. of 7.1 × 10^−4^. Of the distributions fitted to the posterior transfer efficiency (*λ*) values, the Weibull distribution did not visually appear to be a good fit, despite having the lowest AIC value (electronic supplementary material, table S1). The distribution with the second lowest AIC value was Lognormal, with a similar visual fit to the Gamma and Beta distributions (electronic supplementary material, table S1). This distribution was determined to be the best fit, and parameters are available in table 1. It is recommended that the posterior distribution values be used over the fit distributions, but the posterior transfer efficiencies are available in the data associated with this study as well as distribution fits, test statistics and AIC values in the electronic supplementary material, table S1. A strong, positive relationship (*R* = 0.95) was observed between surface and fingertip swabbing efficiency, while other posterior distributions did not have strong relationships (figure 2; electronic supplementary material, figure S2).

**Figure 3.**
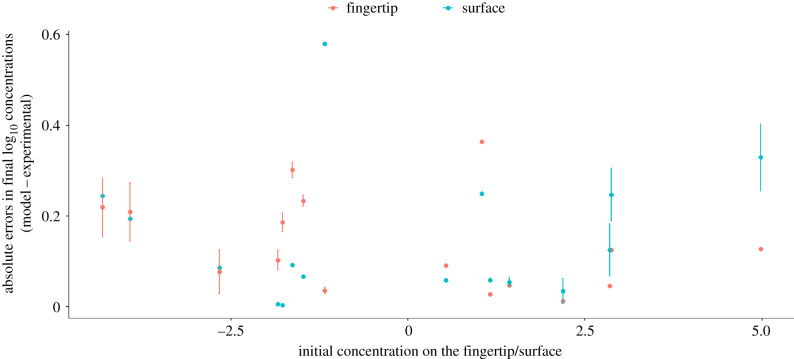
Absolute error (median ± s.d.) in final log_10_ fingertip $\left(C_{k}^{f}\right)$ or surface $\;(C_{k}^{s})$ concentrations as a function of the ratio of before-contact concentration $\left(C_{k-1}^{f}/C_{k-1}^{s}\right)$.

**Figure 4.**
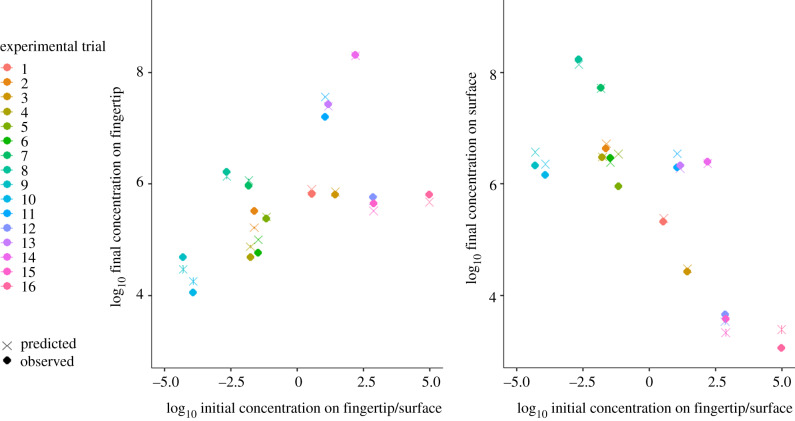
Comparison of log_10_ predicted and observed concentrations on the fingertip and surface after the hand-to-fomite contact ($C_{k}^{f}$ and $C_{k}^{s}$, respectively) for different log_10_ ratios of concentrations on the fingertip and surface before the contact ($C_{k-1}^{f}$ and $C_{k-1}^{s}$, respectively), where error bars for predicted concentrations are the median ± s.d.

There was no significant linear relationship between absolute errors in after-contact concentrations on surface or fingertip and the ratio of starting concentrations (fingertip: *F*_1,14_ = 1.953, *p* = 0.184; surface: *F*_1,14_ = 0.09411, *p* = 0.76) (figure 3). Variability in error and in estimated after-contact concentrations was generally lower when concentrations on the fingertip and surface were similar (figures 3 and 4). This is likely to be due to errors introduced by swabbing efficiency. When those errors are consistent, they affect fingertip and surface concentration in a similar way, not disturbing the ratio of concentrations as much as when one concentration is much larger than the other.

**Figure 5.**
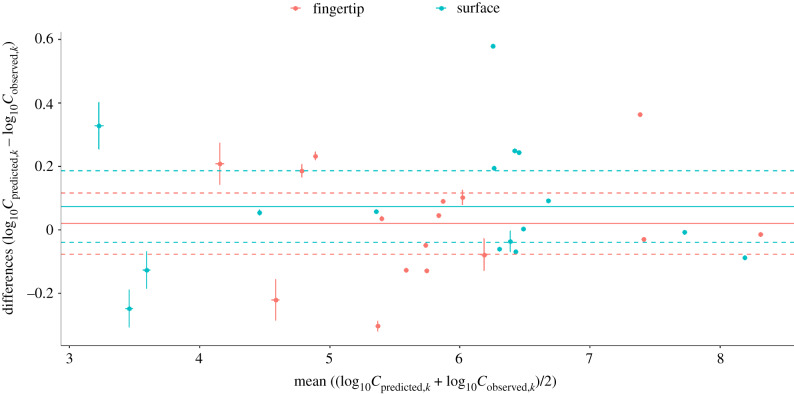
Bland–Altman plot comparing predicted and observed after-contact concentrations on the fingertip $\left(C_{\mathrm{predicted},k}^{f}\right)$ and surface $\left(C_{\mathrm{predicted},k}^{s}\right)$. Mean differences are indicated by the solid line, and 95% confidence intervals (d.f. = 15, *t*_975_ = 2.131) of differences are indicated by the dashed lines. Horizontal and vertical error bars indicate ± s.d.

The 95% confidence intervals (CIs) for differences between median predicted and observed after-contact concentrations overlapped for fingertip and surface, indicating that there is no statistically significant difference in the model's accuracy in predicting the after-contact concentration for the fingertip or surface (figure 5). The average differences between predicted and observed after-contact concentrations for the fingertip and surface were 0.02 log_10_ (95% CI: −0.08, 0.12) and 0.07 log_10_ (95% CI: −0.04, 0.19), respectively. The error in predictions was consistent regardless of the mean of the predicted and observed after-contact concentrations (figure 5). However, greater variability in errors was seen for smaller means of observed and predicted concentrations for both after-contact surface and fingertip concentrations (figure 5).

### 3.4. Quantitative microbial risk assessment application and sensitivity analysis results

**Figure 6.**
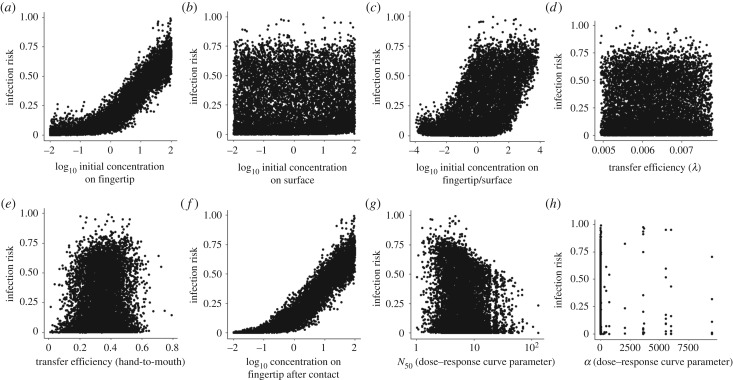
Scatterplots of infection risk versus QMRA parameters for (*a*) log_10_ initial concentration on the fingertip $\left(\text{lo}{\text{g}}_{10}\;C_{k-1}^{f}\right)$, (*b*) log_10_ initial concentration on the surface $\left(\text{lo}{\text{g}}_{10}\;C_{k-1}^{s}\right)$, (*c*) log_10_ initial concentration on fingertip/surface $\left(\text{lo}{\text{g}}_{10}\;C_{k-1}^{f}/C_{k-1}^{s}\right)$, (*d*) transfer efficiency (*λ*), (*e*) transfer efficiency for hand-to-mouth (*TE*_mouth_) and (*f*) log_10_ concentration on the fingertip after the contact $\left(\text{lo}{\text{g}}_{10\;}C_{k}^{f}\right)$; (*g*) dose–response curve parameter *N*_50_ and (*h*) dose–response curve parameter *α*_._

**Figure 7.**
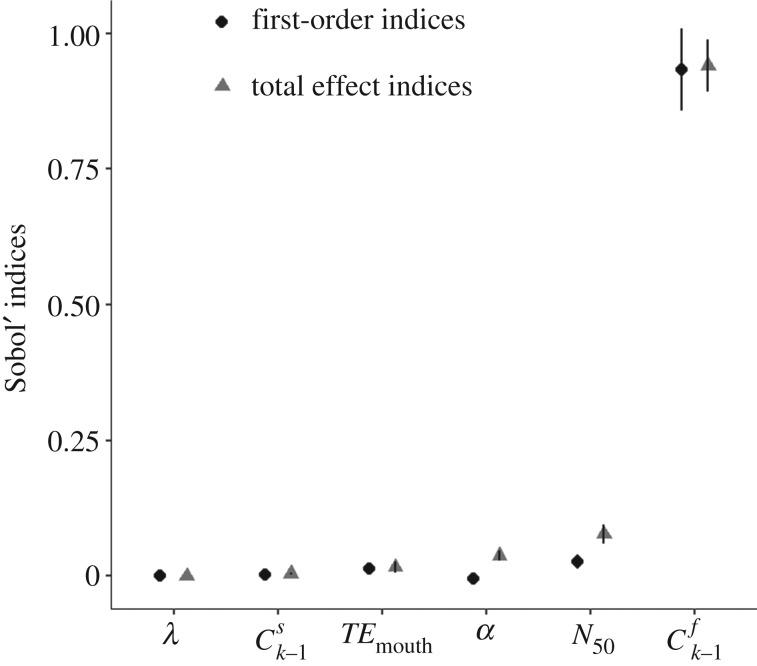
Estimated main and total effect Sobol' indices with 95% confidence intervals for *λ*, *α* (a dose–response curve parameter), *TE*_m__outh_ (hand-to-mouth transfer efficiency), $C_{k-1}^{f}$(concentration on the fingertip before the fingertip-to-surface contact), $C_{k-1}^{s}$(concentration on the surface before the fingertip-to-surface contact) and *N*_50_ (a dose–response curve parameter).

Estimated infection risks from a single contact with the surface ranged from 2.0 × 10^−4^ to 9.9 × 10^−1^ with a median of 1.3 × 10^−1^ and a s.d. of 2.3 × 10^−1^. For estimating these infection risks with equations (2.7)–(2.9), the before-contact log_10_ concentration on the fingertip ($C_{k-1}^{f}$) and the log_10_ concentration on the fingertip after the fingertip-to-surface contact ($C_{k}^{f}$) had the largest Spearman correlation coefficients with estimated infection risk (0.95 and 0.96, respectively) (figure 6*a*,*f*; electronic supplementary material, table S2). While the before-contact log_10_ concentration on the surface did not appear to have a strong relationship with infection risk (figure 6*b*; electronic supplementary material, table S2), the log_10_ ratios of before-contact concentrations on the fingertip and surface $\left(C_{\mathrm{observed},k-1\;}^{f}/C_{\mathrm{observed},k-1}^{s}\right)$ did have a strong relationship with infection risk, with a Spearman correlation coefficient of 0.60 (figure 6*c*; electronic supplementary material, table S2). Transfer efficiencies for both surface-to-fingertip and fingertip-to-mouth and the dose–response curve parameters (*N*_50_ and *α*) did not have strong relationships with infection risk (figure 6*d,e*,*g*,*h*; electronic supplementary material, table S2). The before-contact concentration of virus on the fingertip explained the most variance in infection risk, having the largest estimated main and total effect Sobol' indices (figure 7). The next most influential parameter was the dose–response curve parameter, *N*_50_ (figure 7).

## 4. Discussion

### 4.1. Key findings and generalizability

This study supports the hypothesis that transfer of viruses between a contaminated fingertip and surface occurs as a function of a concentration gradient, with transfer occurring in both directions. This was demonstrated through the fit of a logistic curve to recoverable transfer efficiencies as a function of log_10_-transformed ratios of before-contact concentrations on the fingertip and surface (figure 1). Furthermore, the study shows that concentrations after a fingertip-to-surface contact can be predicted by a viral exposure model framework that assumes a gradient of transfer, where, on average, the model over-estimates after-contact concentration on the fingertip by 0.02 log_10_ (figure 5). Generally, viruses appear to stay on their original surfaces, with a fraction transferring from the more to the less contaminated object, confirmed here by the posterior distribution for transfer efficiency and by the fit of a logistic curve to experimental data (figures 1 and 2).

The gradient transfer model allows for modelling transfer in both directions, as it assumes transfer occurs from higher to lower concentrations. The microbial transfer model's use of transfer efficiency (*λ*) is similar to the traditional use of ‘transfer efficiency' in scenarios where recipient surfaces are uncontaminated; the meaning differs from the traditional definition of ‘transfer efficiency' (fraction of total microbes transferred from one surface to another) when both donor and recipient surfaces are contaminated. When only one surface is contaminated, transfer efficiency is the fraction of virus transferred from the contaminated surface to another and represents a fraction of the total virus available for transfer (equations (2.4) and (2.5)). When both surfaces are contaminated, transfer efficiency in the model represents the fraction of viruses on each surface individually as opposed to the total amount of virus available for transfer (equations (2.4) and (2.5)). This difference and resulting differences in exposure model estimates for repetitive contact scenarios should be considered in future research.

Calculating transfer efficiencies is complicated by uncertainty and variation in estimates of virus contamination on surfaces. The number of total quantified virus particles before a fingertip-to-fomite contact often differs from the total number after a fingertip-to-fomite contact, sometimes resulting in transfer efficiencies over 100% [9]. In this study, ABC alleviated this issue by assuming that differences in total detected virus before and after a fingertip-to-fomite contact were attributed to differences in fingertip and surface swabbing efficiencies. If swabbing efficiencies were the same for the fingertip and the surface, it would be expected that the final viral count before and after the contact would remain the same. However, this was not experimentally observed, and differences in posterior distributions for fingertip and surface swabbing efficiency (figure 2) suggest that they were not the same. Experimental studies have also demonstrated the influence of surface material on viral recovery efficiencies using swabs [38,39].

Fingertip and surface swabbing efficiencies in the posterior distributions had a strong positive relationship, where a linear fit of posterior surface swabbing efficiencies versus fingertip swabbing efficiencies had an *R*^2^ of 0.90 and a slope of 0.82 (electronic supplementary material, figure S2). The slope being close to 1 implies that large differences in swabbing efficiencies between the fingertip and the surface result in less agreement between observed and expected transfer efficiencies. Mechanistically, this is explainable by the impact that swabbing efficiencies have on the estimated concentration of present virus (not just detected). For example, similar swabbing efficiencies result in estimated concentrations of present virus that are closer to the observed concentrations than when swabbing efficiencies are dissimilar. Because the observed concentrations are fixed at their measured values, estimated concentrations of present virus are necessarily higher when small swabbing efficiencies are assumed relative to when large swabbing efficiencies are assumed (equations (2.4) and (2.5)). As the Euclidean distance calculation minimizes the differences between predicted and observed values for both surfaces and fingers collectively, swabbing efficiencies must be similar in value to balance discrepancies between total before- and total after-contact concentrations (equation (2.6)). Nevertheless, a mechanistic explanation behind the exact relationship remains unclear.

We demonstrate the use of posterior distributions obtained through ABC in extending a specific experimental study by relating it to health risk estimates. While this method is relatively new to the exposure science field [21], a common limitation of an ABC rejection sampler, as used in this study, is that it can get stuck in local minima and that it does not explore the parameter space very efficiently. While the posterior distributions of swabbing efficiencies and transfer efficiencies are reasonable, other methods should be explored in future research, such as an ABC-sequential Monte Carlo approach, as used by Toni *et al*. [28]. Additionally, we did not have prior information regarding theoretical distributions for swabbing efficiencies, and only limited information for transfer efficiencies relevant to the experimental design in this study (contact between two contaminated surfaces), which led us to use uninformative priors (uniform distributions) for these parameters. Future development of this method for exposure science applications should include investigating the influence of prior distribution choices on the posterior estimates and estimated health outcomes.

The QMRA application in this study implies that dose–response curve parameters (figure 7) and after- and before-contact concentrations on fingertips (figures 6 and 7; electronic supplementary material, table S2) are the most influential on infection risk. This is consistent with the results of prior QMRA work, which frequently shows the importance of microbial contamination on surfaces [16,26] and dose–response parameters [40]. While transfer efficiency did not appear to have a large influence on infection risk in this study relative to other model parameters (figures 6 and 7; electronic supplementary material, table S2), the posterior distribution included small transfer efficiency values, which may explain why transfer efficiency had little impact. This is anticipated in simple, linear models, like the one evaluated in this study, where small numbers will have a small influence. Transfer efficiency could be larger for other organisms or surface types, supporting further research defining these posterior distributions for a variety of organisms, surface types and exposure models.

For the dataset used in this study, the model more accurately explained changes in concentration when the starting concentrations on the fingertip and surface were similar (figure 4), and greater variability in predictions were observed when one of the surface concentrations (fingertip or surface) was small relative to the other (figures 4 and 5). Other datasets and mechanistic models that may better explain or predict concentration changes when the before-contact concentration on one object is smaller than the other should be evaluated.

### 4.2. Limitations

Swabbing efficiencies specific to our organisms of interest on a porous ceramic tile were unavailable to our knowledge. This led to the selection of uninformative priors for swabbing efficiency parameters (table 1). For MS2, one of the organisms used in this study, the median fractions of detectable MS2 for cotton, antistatic and polyester swabs with different eluent types (saline, Ringer's, viral transport media, acid/base) sampled from stainless steel and plastic surfaces ranged from 7 × 10^−2^ to 3.8 × 10^−1^ [41]. The median of the surface swabbing efficiency posterior distribution in this study (median = 5.6 × 10^−1^, s.d. = 2.0 × 10^−1^) was considerably larger than this range. In a study in which rotavirus was used, the recovery of virus inoculated on the hand was reported as approximately 82% (8.2 × 10^−1^) [42]. However, this recovery efficiency is not specific to swabs used in this study, but rather the use of undiluted tryptose phosphate broth, 20% tryptose phosphate broth and Earle balanced salt solution in a vial inverted on the fingertip [42]. The median and s.d. of the posterior distribution for fingertip swabbing efficiency in this study were 7.1 × 10^−1^ and 2.4 × 10^−1^, respectively. Despite the lack of prior knowledge about swabbing efficiencies relevant to the experimental scenario in this study, we were still able to ‘learn', or see a notable difference between posterior and prior distributions, for fingertip and surface swabbing efficiencies. This allowed us to account for uncertainty in swabbing efficiencies, yielding a narrow range for the posterior transfer efficiency distribution.

The results of this study are derived from experimental data using two phages (MS2 and *Φ*X174), which may not be generalizable to other organism combinations. The use of the two phages MS2 and *Φ*X174 was driven by other studies demonstrating the use of safe, easy to use, surrogate viruses for common enteric pathogens [7,9,12,13]. However, phage fate and transport processes may differ from enteric viruses. The study used both viruses simultaneously in a single assay to understand and quantify the overall exchange of viruses from one object to another. If one phage had been used, only the net effect would have been captured. Furthermore, viruses were inoculated at high concentrations, above what may be real-world concentrations. High concentrations were used in order to avoid left-censored data. Additionally, while the participant practised achieving a contact force between 700 and 1500 g on the scale, some contacts were outside of this range and point values for the 1 second contact were not consistently observed by the participant. Future studies should explore alternative approaches for achieving consistent contact forces or recording maximum contact force applied during testing (i.e. videography, instrument value recording).

## 5. Conclusion

We used experimental and computational methods to evaluate a hypothesis that microbes follow a gradient of transfer for fingertip-to-surface contacts. Experimentally, this behaviour was demonstrated, where recoverable transfer efficiencies versus ratios of before-contact virus concentrations on the fingertip and surface were described by a logistic curve (figure 1). Using an ABC approach, it was demonstrated that a microbial exposure model that assumes a gradient of transfer was able to describe experimentally measured final viral concentrations on the fingertip and surface with less than 1 log_10_ of error, offering insights into swabbing efficiencies and experimental transfer efficiencies. Posterior distributions gleaned from the ABC approach can be used in future exposure modelling applications.

## Supplementary Material

Figure S1. Dose response curve fit with 95% and 99% confidence intervals, where triangle symbols represent experimental data

## Supplementary Material

Figure S2.

## Supplementary Material

Table S1. Comparison of candidate distribution fits to the posterior transfer efficiency values and goodness of fit test results*

## Supplementary Material

Table S2. Spearman correlation coefficients

## Data accessibility

Code and data are available at https://doi.org/10.5518/801.
