# Supplementary figures and images for: Evaluating a transfer gradient assumption in a fomite-mediated microbial transmission model using an experimental and Bayesian approach
Source: J R Soc Interface. 2020 Jun 24;17(167):20200121. doi: 10.1098/rsif.2020.0121 (PMC7328381; doi:10.1098/rsif.2020.0121)

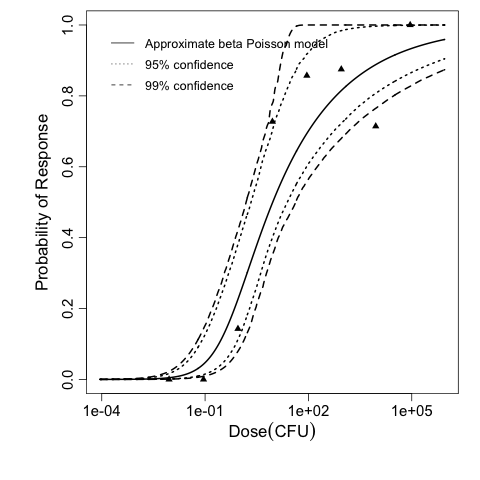

Supplement: Figure S1. Dose response curve fit with 95% and 99% confidence intervals, where triangle symbols represent experimental data [file rsif20200121supp1.png]

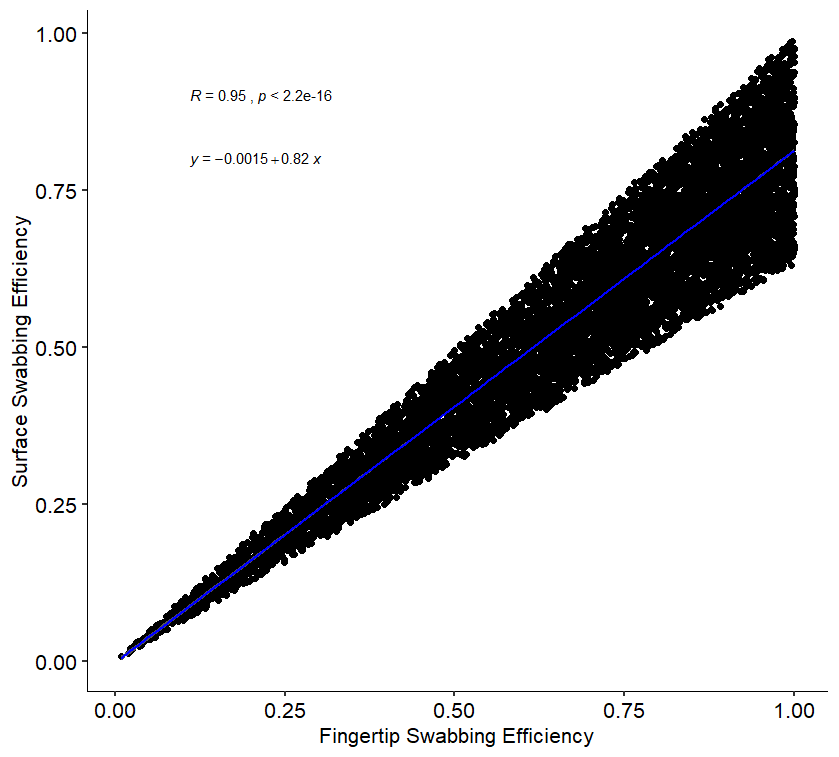

Supplement: Figure S2. [file rsif20200121supp2.png]
