# Supplementary material for: Evaluating a transfer gradient assumption in a fomite-mediated microbial transmission model using an experimental and Bayesian approach
Source: J R Soc Interface. 2020 Jun 24;17(167):20200121. doi: 10.1098/rsif.2020.0121 (PMC7328381; doi:10.1098/rsif.2020.0121)
Supplement: Table S1. Comparison of candidate distribution fits to the posterior transfer efficiency values and goodness of fit test results* [file rsif20200121supp3.docx]

**Table S1.** Comparison of candidate distribution fits to the posterior transfer efficiency values and goodness of fit test results*

| **Distribution** | **Parameters** | **Kolmogorov-Smirnov test statistic** | $\boldsymbol{\chi}^{\boldsymbol{2}}$ **test statistic** | **AIC** | **Figure of Fit** |
| --- | --- | --- | --- | --- | --- |
| Lognormal | Meanlog =  -5.0732843  Sdlog = 0.1132161 | 0.04615422 | 962.638 | -116652 | 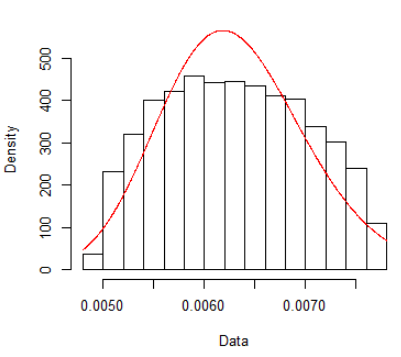 |
| Gamma | Shape: 78.55785  Rate: 12465.71421 | 0.04342722 | 943.272 | -116676.8 | 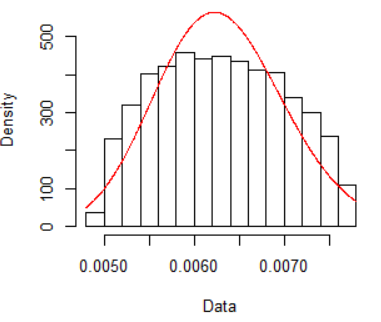 |
| Beta | Shape 1: 78.05378  Shape 2: 12307.48574 | 0.04333077 | 942.8318 | -116677.2 | 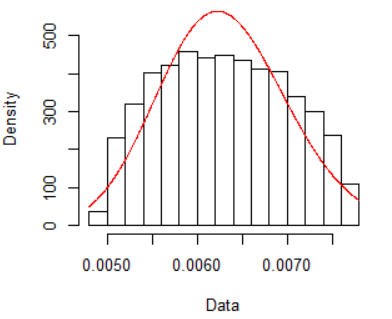 |
| Weibull | Shape: 9.810273133  Scale: 0.006621803 | 0.05827289 | 1360.087 | -116165 | 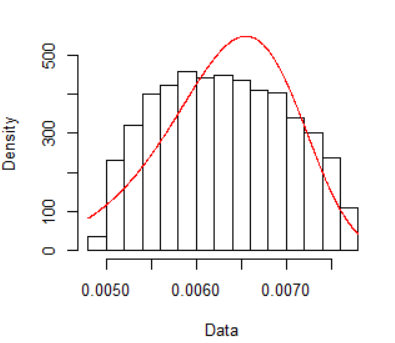 |

* Distributions should be left- and right-truncated at 0 and 1, due to physical limitations of transfer efficiencies, defined here as a fraction of total virus transferred.
