# Supplementary material for: Evaluating a transfer gradient assumption in a fomite-mediated microbial transmission model using an experimental and Bayesian approach
Source: J R Soc Interface. 2020 Jun 24;17(167):20200121. doi: 10.1098/rsif.2020.0121 (PMC7328381; doi:10.1098/rsif.2020.0121)
Supplement: Table S2. Spearman correlation coefficients [file rsif20200121supp4.docx]

**Table S2.** Spearman correlation coefficients

| Parameter | Variable | Spearman correlation coefficient with infection risk |
| --- | --- | --- |
| log_10_ concentration on fingertip after fingertip-to-surface contact | $\log_{10}C_{k-1}^{f}$ | 0.96 |
| log_10_ initial concentration on the fingertip | $\log_{10}C_{k-1}^{f}$ | 0.95 |
| log_10_ initial concentration on the fingertip/surface | $\log_{10}\frac{C_{k-1}^{f}}{C_{k-1}^{s}}$ | 0.60 |
| dose-response curve parameter | $N_{50}$ | -0.16 |
| transfer efficiency  (fingertip-to-mouth) | $\mathrm{TE}_{\mathrm{mouth}}$ | 0.12 |
| log_10_ initial concentration on the surface | $\log_{10}C_{k-1}^{s}$ | 0.10 |
| dose-response curve parameter | $\alpha$ | -0.04 |
| transfer efficiency  (fingertip-to-surface or surface-to-fingertip) | λ | 0.03 |
